# Supplementary material for: Biomaterial-based scaffold for in situ chemo-immunotherapy to treat poorly immunogenic tumors
Source: Nat Commun. 2020 Nov 10;11:5696. doi: 10.1038/s41467-020-19540-z (PMC7655953; doi:10.1038/s41467-020-19540-z)
Supplement: Supplementary file 1 — Supplementary Information [file 41467_2020_19540_MOESM1_ESM.pdf]

Supplementary Information for

Biomaterial-Based Scaffold for In Situ Chemo-Immunotherapy to Treat Poorly Immunogenic Tumors

Hua Wang[1,2], Alexander J. Najibi[1,2], Miguel C. Sobral[1,2], Bo Ri Seo[1,2], Jun Yong Lee[1,2,3], David Wu[1,2], Aileen Weiwei Li[1,2], Catia S. Verbeke[1,2], David J. Mooney[1,2]*

[1]Harvard John A. Paulson School of Engineering and Applied Sciences, Harvard University, Cambridge, Massachusetts 02138, USA. [2]Wyss Institute for Biologically Inspired Engineering, Cambridge, Massachusetts 02138, USA. [3]Department of Plastic and Reconstructive Surgery, College of Medicine, The Catholic University of Korea, Seoul, Republic of Korea.

*email: mooneyd@seas.harvard.edu

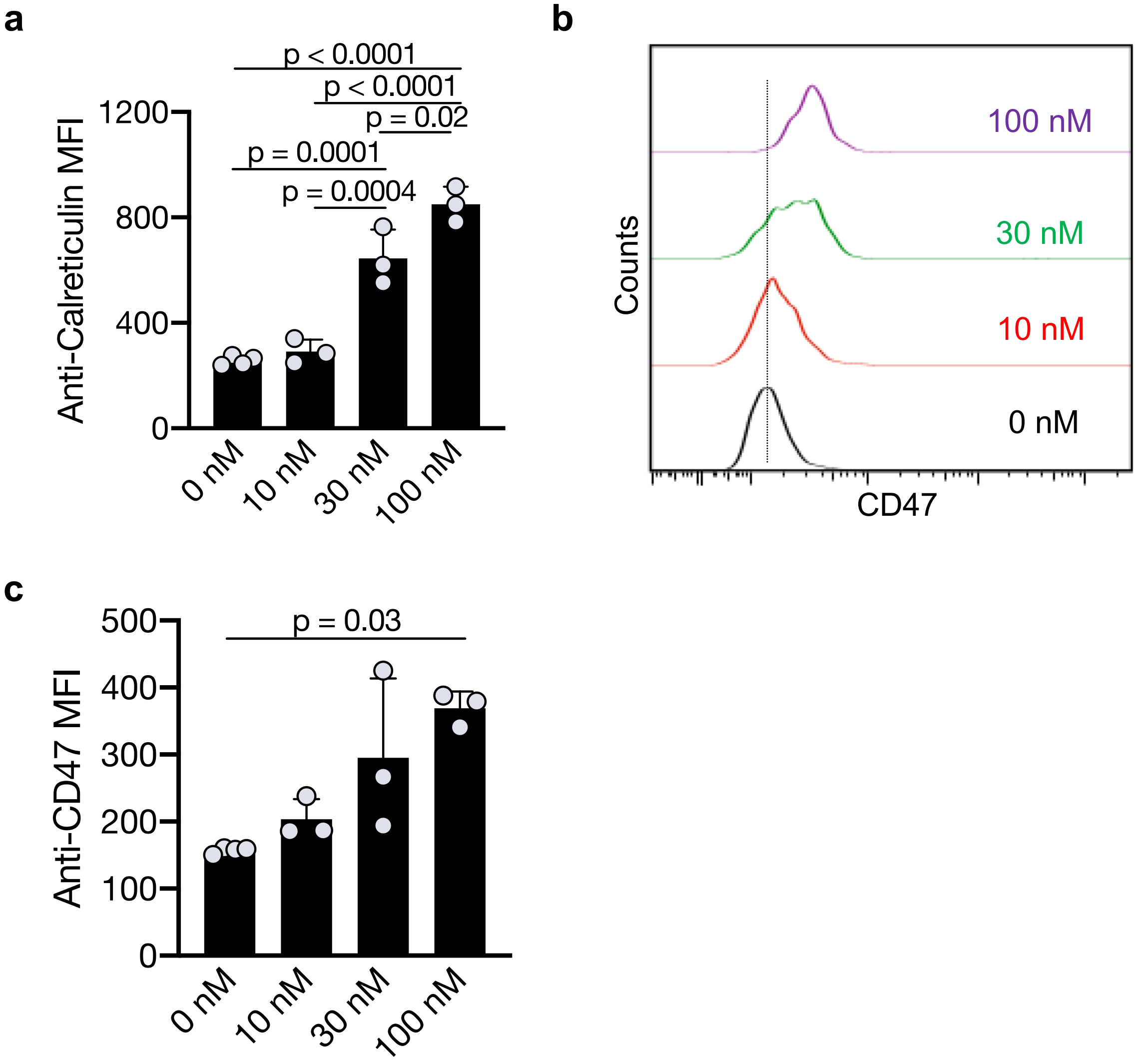

Supplementary Figure 1. **Dox induces immunogenic death of 4T1 cells in vitro**. (a) Mean Alexa Fluor 647 fluorescence intensity of 4T1 cells after treatment with different concentrations of Dox for 24 h and incubation with Alexa Fluor 647-conjugated anti-calreticulin for 20 min. (b) Representative flow cytometry histograms of 4T1 cells after treatment with different concentrations of Dox for 24 h and incubation with FITC-conjugated anti-CD47 for 20 min. Live/dead cell gating has already been performed. (c) Mean FITC fluorescence intensity of 4T1 cells with the same treatment in (b). For a and c, data are presented as mean ± SD; n = 4 biologically independent samples per group. Statistical analysis was performed using ANOVA with Tukey's (a) or Kruskal-Wallis with Dunn's (c) post hoc test. Source data are provided as a Source Data file.

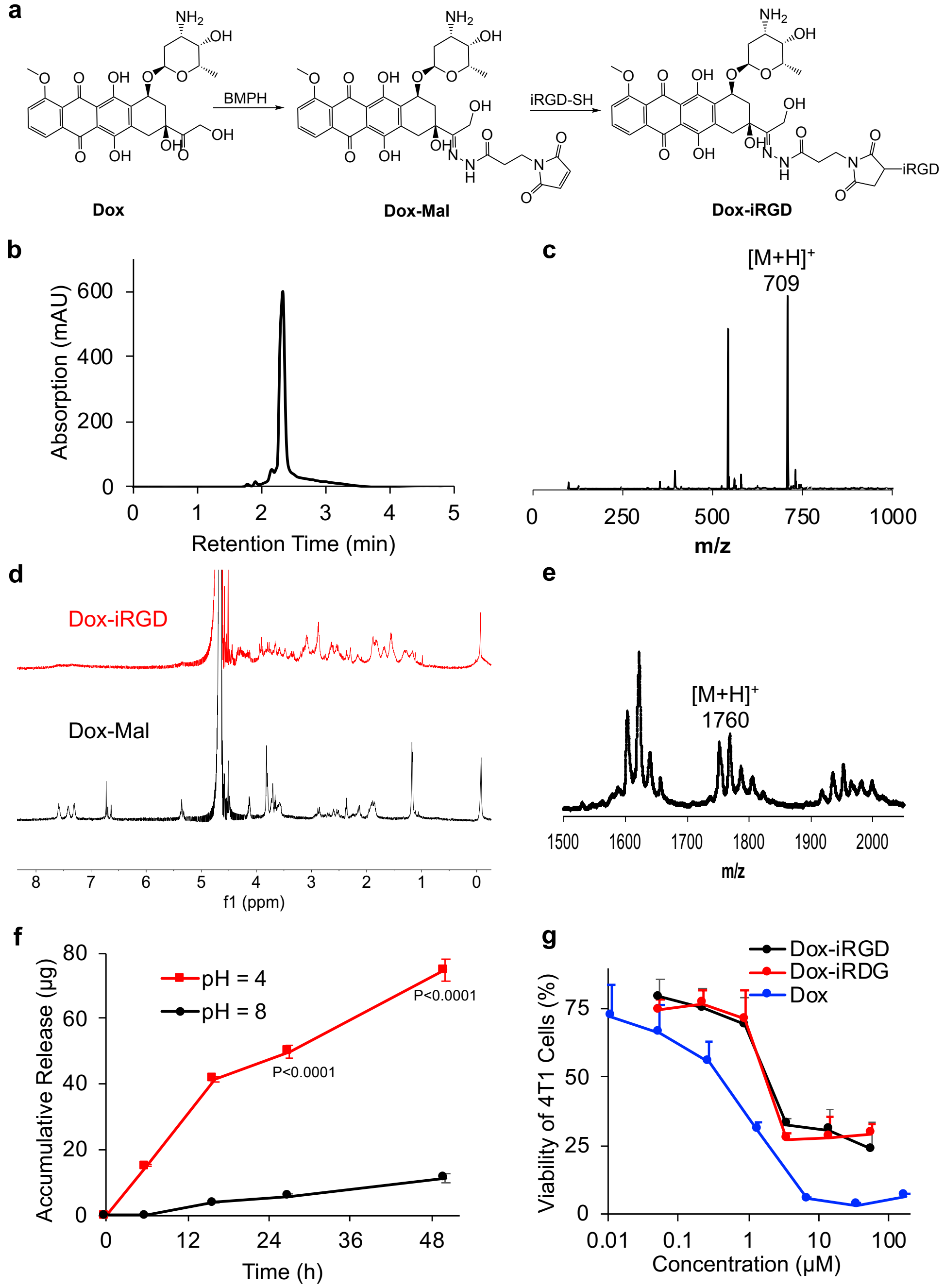

Supplementary Figure 2. Synthesis and characterization of Dox-iRGD. (a) Synthetic route of Dox-iRGD. High-performance liquid chromatography (b) and mass spectrum (c) of Dox-Mal, at the detection wavelength of 254 nm. (d) ^{1}H NMR spectra of Dox-Mal and Dox-iRGD, respectively. (e) MALDI spectrum of Dox-iRGD. (f) Degradation profiles of Dox-iRGD into Dox at pH 4 and 8, respectively. Data are presented as mean ± SD; n = 4 biologically independent samples per group. Statistical analysis was performed using two-tailed t tests. (g) Viability of 4T1 cells after incubation with different concentrations of Dox-iRGD, Dox-iRDG, and Dox, respectively, for 48 h. Data are presented as mean ± SD; n = 4 biologically independent samples per group. Source data are provided as a Source Data file.

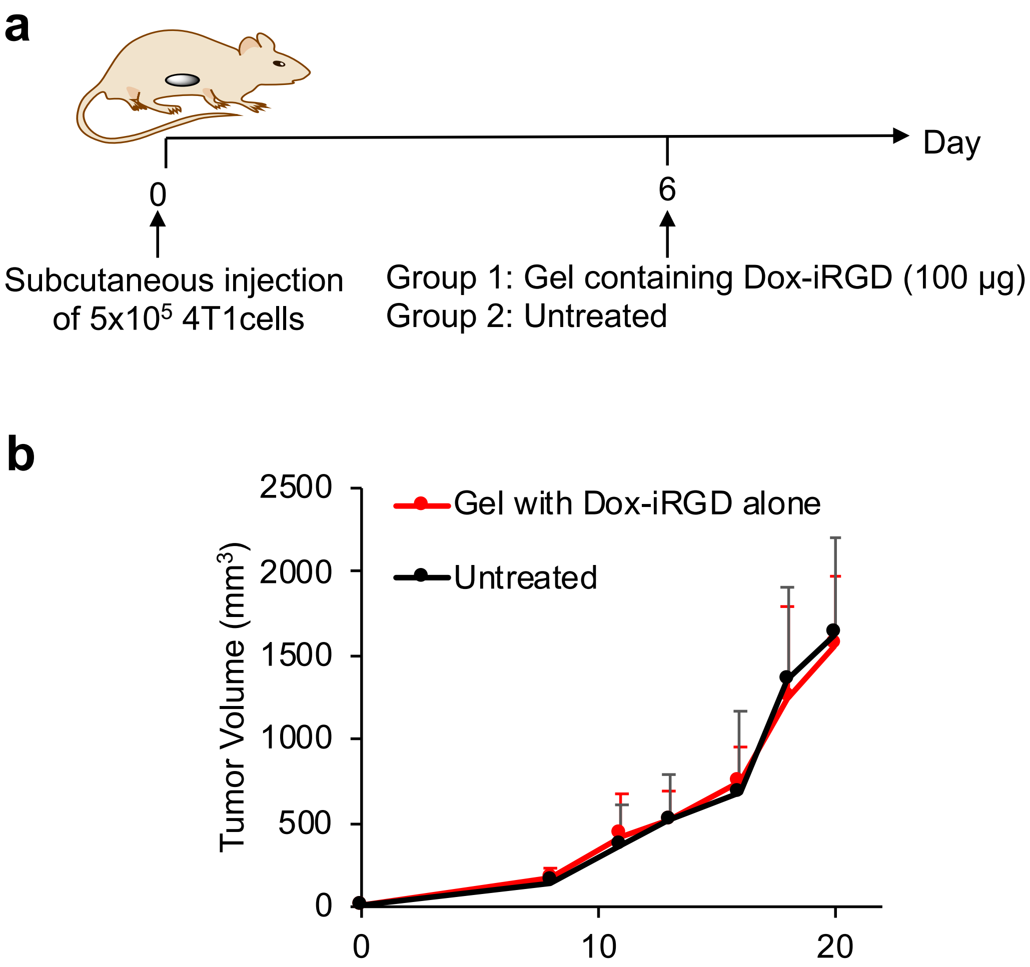

Supplementary Figure 3. **Pore-forming gels containing Dox-iRGD alone do not show therapeutic benefit against 4T1 tumors**. (a) Timeframe of efficacy study. Gels were peritumorally injected when the tumors grew to ~6-7 mm. The drug dose is described in Dox equivalent. (b) Tumor growth profiles for each group. Data are presented as mean ± SD; n = 5 biologically independent animals per group. Source data are provided as a Source Data file.

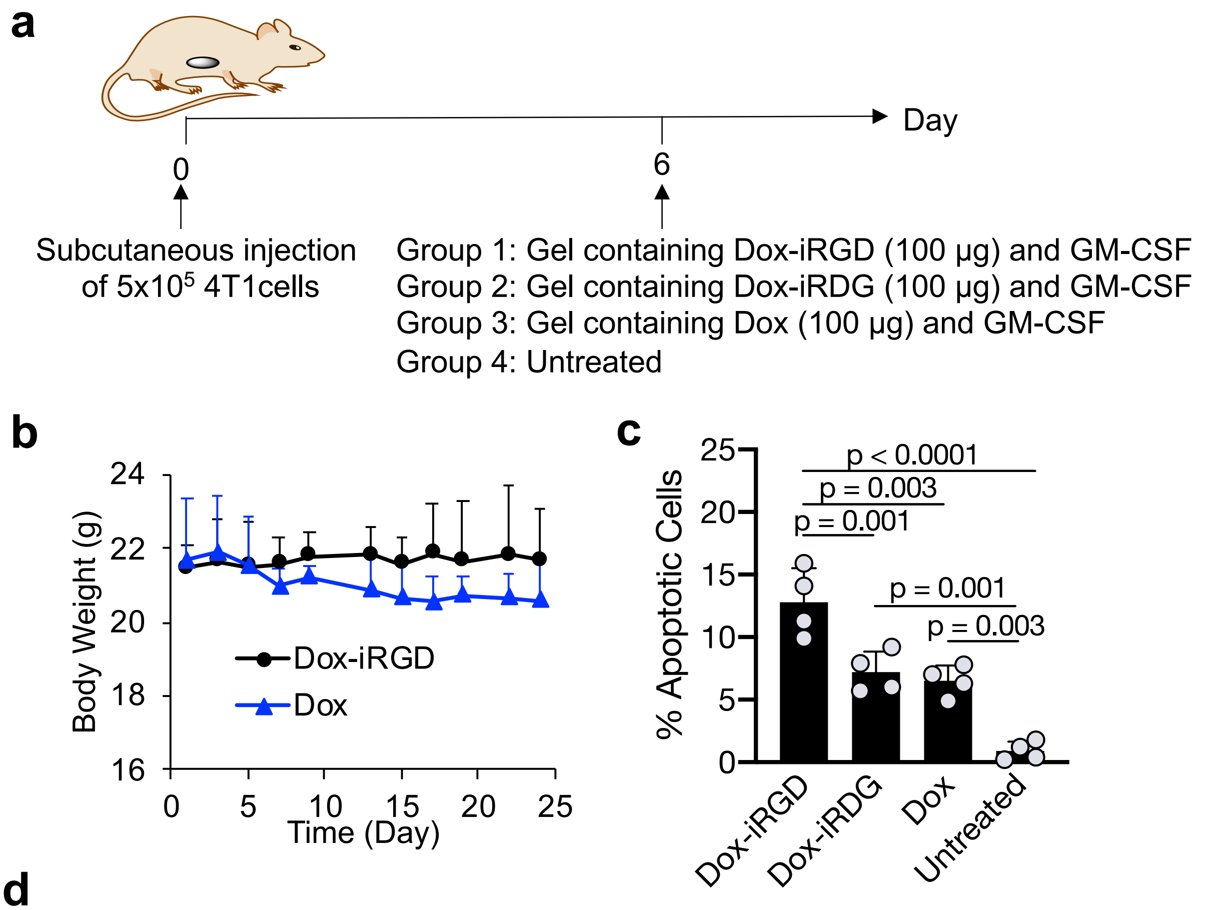

d

Acute toxicity	Dox	Dox-iRGD	Dox-iRDG	Untreated
Bone marrow: Decreased cellularity	+/+++ (5/5)	- (0/5)	- (0/5)	- (0/5)

Supplementary Figure 4. **Pore-forming gels containing Dox-iRGD improve antitumor efficacy and reduce systemic toxicity compared to gels containing Dox**. (a) Timeframe of efficacy study. Gels were peritumorally injected when the tumors grew to ~6-7 mm. The drug dose is described in Dox equivalent. (b) Average body weight of mice treated with gels containing Dox-iRGD and Dox, respectively. Data are presented as mean ± SD; n = 5 biologically independent animals per group. (c) Percentage of apoptotic tumor cells at 4 days post gel injection, as determined by the TUNEL assay. Data are presented as mean ± SD; n = 5 biologically independent animals per group. Statistical analysis was performed using ANOVA with Tukey's post hoc test. (d) Toxicity evaluation of gels loaded with Dox, Dox-iRGD, or Dox-iRDG in BALB/c mice. + (mild); +++ (marked); - (negative). Source data are provided as a Source Data file.

a

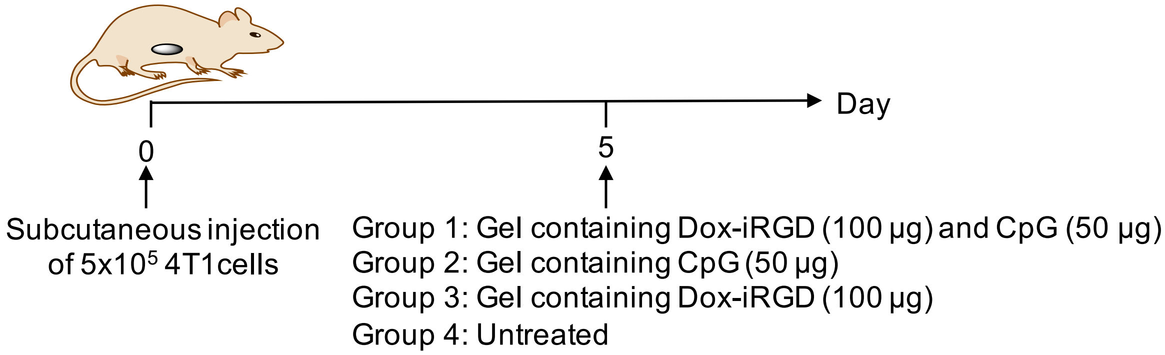

b

Group Description	Median Survival (day)	Survival Increase (day)	%Survival Increase
Dox-iRGD+CpG	39	12	44.4
CpG	32	5	18.5
Dox-iRGD	31	4	14.8
Untreated	27	--	--

Supplementary Figure 5. **In situ gel vaccine containing GM-CSF, Dox-iRGD (100 µg) and CpG (50 µg) prolongs animal survival.** (a) Time frame of efficacy study. GM-CSF was incorporated in all gels. (b) Summary of median survival for each group and increase of median survival in treatment groups in comparison to untreated group.

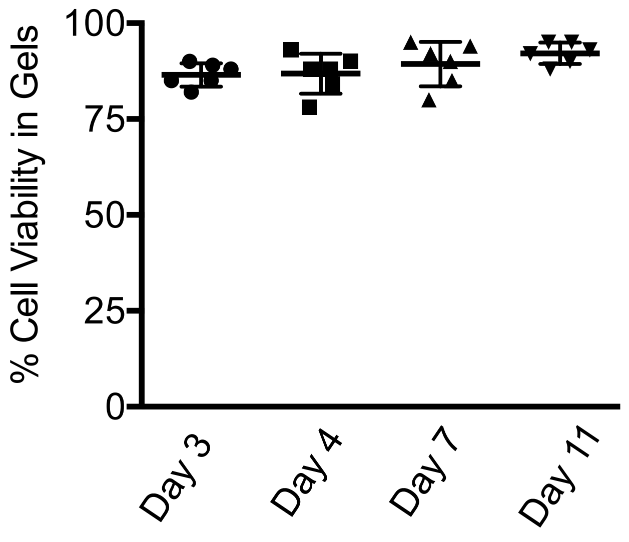

Supplementary Figure 6. Viability of Cells in gels that were harvested at different times post peritumoral injection. Data are presented as mean ± SD; n = 6 biologically independent animals per group. Gels were loaded with GM-CSF, Dox-iRGD (200 µg), and CpG (100 µg). Source data are provided as a Source Data file.

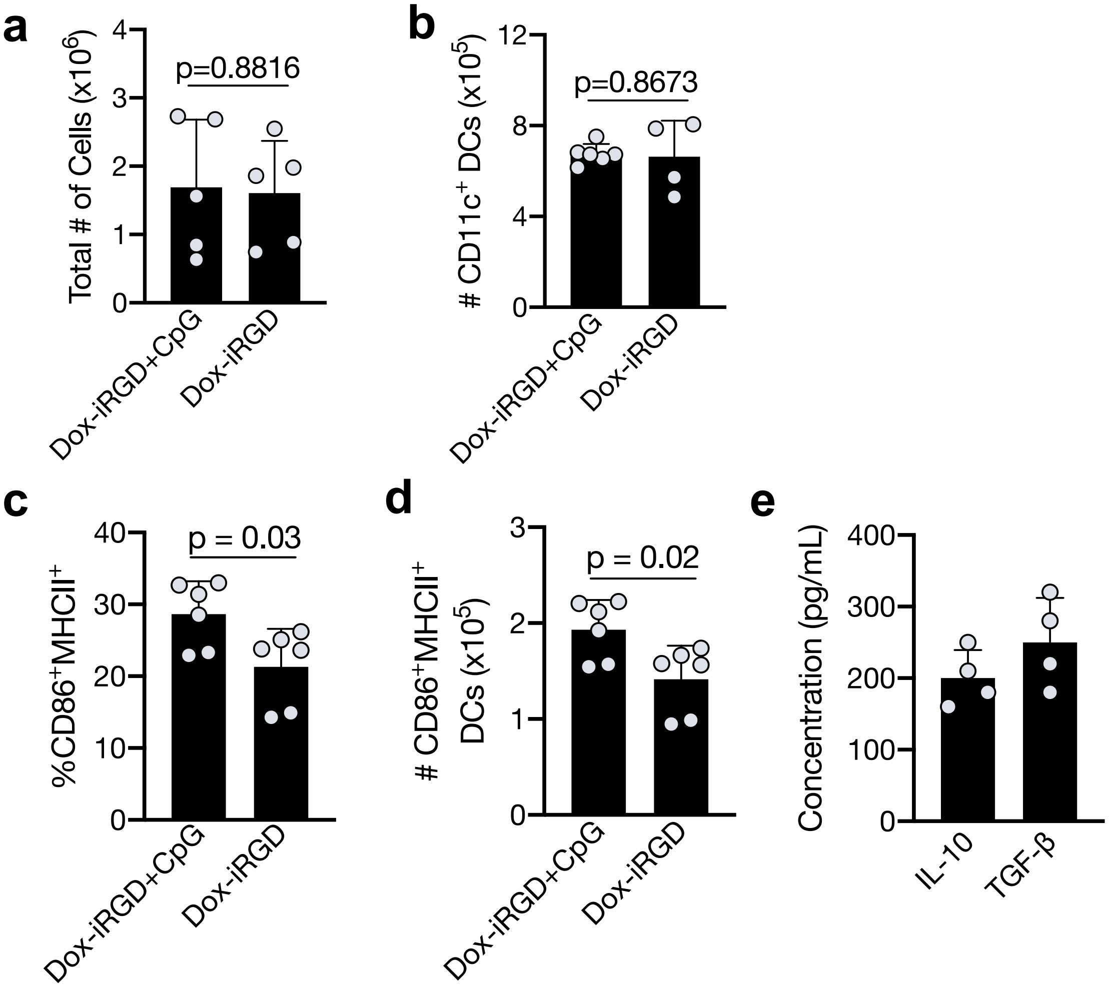

Supplementary Figure 7. **Pore-forming gels containing GM-CSF, Dox-iRGD and CpG recruit and activate DCs**. (a) Total number of recruited cells in gels at 4 days post injection of gels containing Dox-iRGD (200 μg) and CpG (100 μg) or Dox-iRGD alone. GM-CSF was incorporated in all gels. (b) Number of CD11c$^+$ DCs in gels at 4 days post gel injection. (c) Percentage of CD86$^+$MHCII$^+$ cells among CD11c$^+$ DCs in gels. (d) Number of CD86$^+$MHCII$^+$ DCs in gels. (e) Concentration of IL-10 and TGF-β in gels loaded with GM-CSF, Dox-iRGD, and CpG at 4 days post subcutaneous injection. For a-e, data are presented as mean ± SD; n = 6 biologically independent animals per group. Statistical analysis was performed using two-tailed t tests. Source data are provided as a Source Data file.

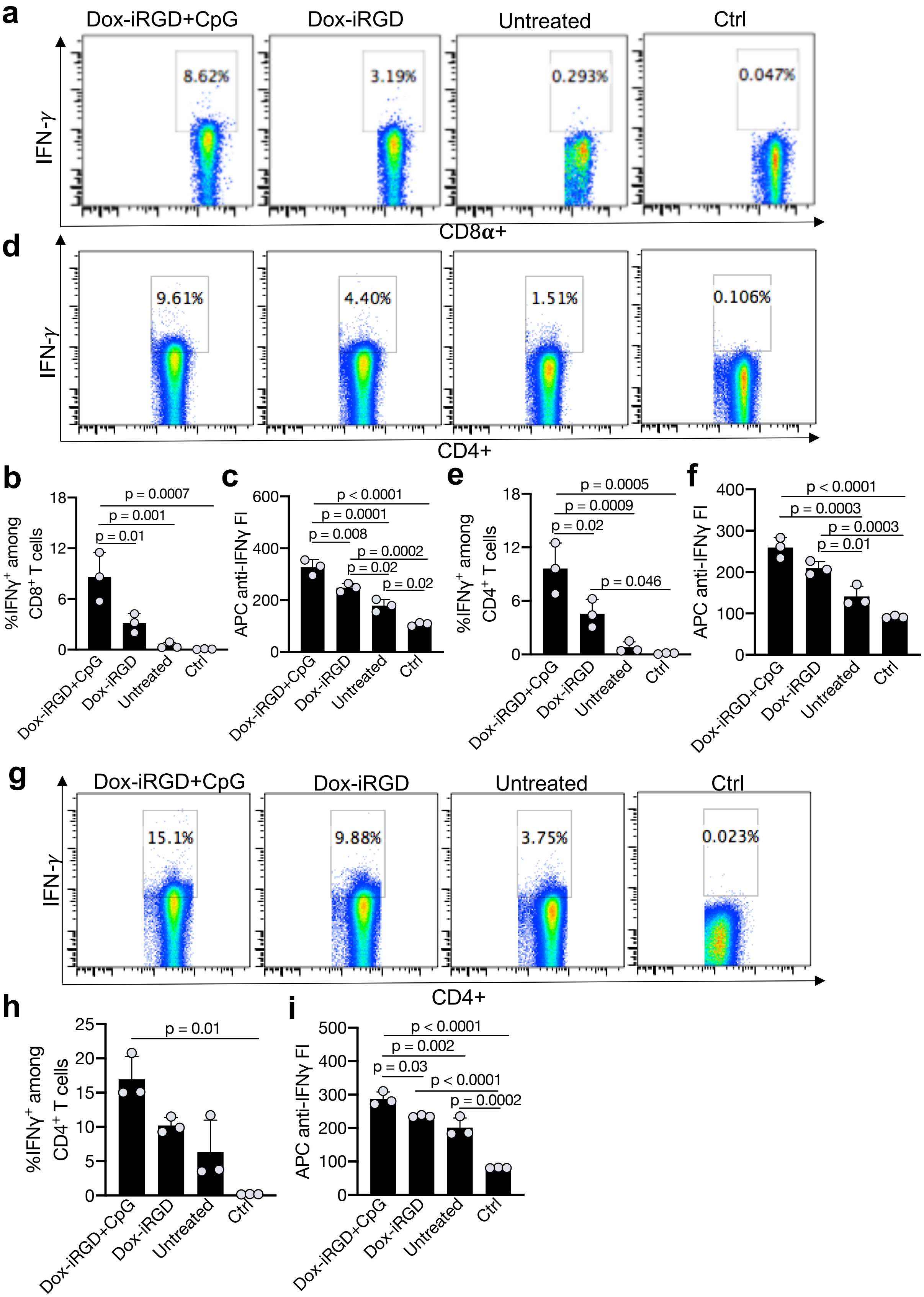
a
Dox-iRGD+CpG
Dox-iRGD
Untreated
Ctrl
IFN-γ
8.62%
3.19%
0.293%
0.047%
CD8α+
d
IFN-γ
9.61%
4.40%
1.51%
0.106%
CD4+
b
%IFNγ+ among CD8+ T cells
p = 0.0007
p = 0.001
p = 0.01
c
APC anti-IFNγ FI
p < 0.0001
p = 0.0001
p = 0.008
p = 0.0002
p = 0.02
p = 0.02
e
%IFNγ+ among CD4+ T cells
p = 0.0005
p = 0.0009
p = 0.02
p = 0.046
f
APC anti-IFNγ FI
p < 0.0001
p = 0.0003
p = 0.0003
p = 0.01
g
Dox-iRGD+CpG
Dox-iRGD
Untreated
Ctrl
IFN-γ
15.1%
9.88%
3.75%
0.023%
CD4+
h
%IFNγ+ among CD4+ T cells
p = 0.01
i
APC anti-IFNγ FI
p < 0.0001
p = 0.002
p = 0.03
p < 0.0001
p = 0.0002

Supplementary Figure 8. **Pore-forming gels containing GM-CSF, Dox-iRGD and CpG generate potent systemic tumor-specific CTL responses**. GM-CSF was incorporated in all gels. (a) Representative IFN-γ versus CD8 plots of splenocytes at 4 days post injection of gels containing Dox-iRGD (200 µg) and CpG (100 µg) or Dox-iRGD alone. GM-CSF was incorporated in all gels. (b) Percentage of IFN-γ^+ cells and (c) mean APC anti-IFN-γ fluorescence intensity among $CD8^+$ T cells. (d) Representative IFN-γ versus CD4 plots of splenocytes in different groups. (e) Percentage of IFN-γ^+ cells and (f) mean APC anti-IFN-γ fluorescence intensity among $CD4^+$ T cells in spleens of different groups. (g) Representative IFN-γ versus CD4 plots of cells isolated from tumor-draining lymph nodes (tdLNs). (h) Percentage of IFN-γ^+ cells and (i) mean APC anti-IFN-γ fluorescence intensity among $CD4^+$ T cells in tdLNs. For b-f and h-i, data are presented as mean ± SD; n = 3 biologically independent animals per group. Statistical analysis was performed using ANOVA with Tukey's LSD post hoc test (b,c,e,f,i) and Kruskal-Wallis with Dunn's post hoc test (h). Source data are provided as a Source Data file.

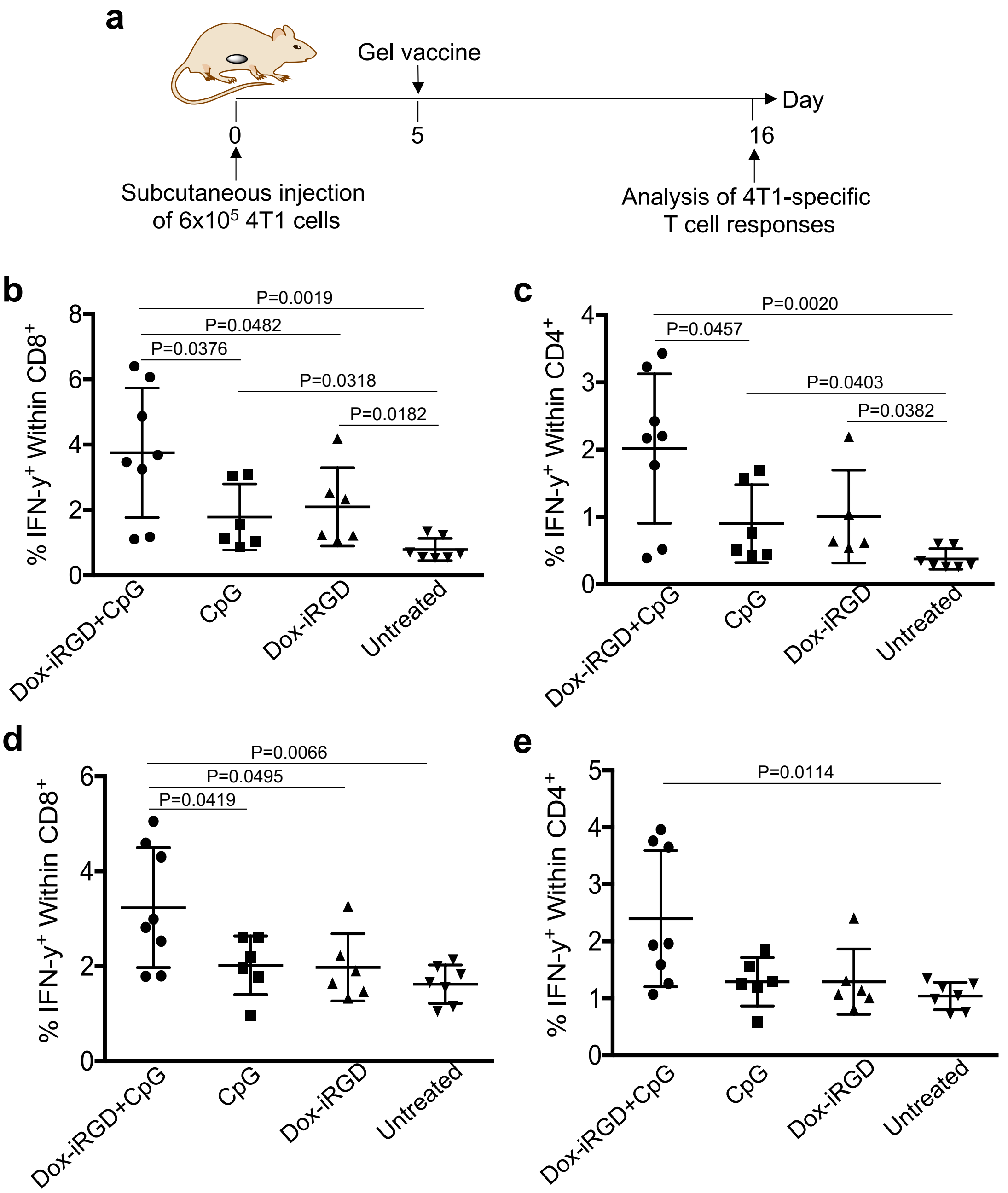

Supplementary Figure 9. **Pore-forming gels containing GM-CSF, Dox-iRGD and CpG generate potent systemic tumor-specific CTL responses at 11 days post injection**. (a) Time frame of animal study. GM-CSF was incorporated in all gels. (b) Percentage of IFN-γ^+ cells among $CD8^+$ T cells in tdLNs at 11 days post injection of gels. (c) Percentage of IFN-γ^+ cells among $CD4^+$ T cells in tdLNs at 11 days post injection of gels. (d) Percentage of IFN-γ^+ cells among $CD8^+$ T cells in spleens at 11 days post injection of gels. (e) Percentage of IFN-γ^+ cells among $CD4^+$ T cells in spleens at 11 days post injection of gels. For b-e, data are presented as mean ± SD; n = 8 biologically independent animals for Dox-iRGD + CpG group, n = 6 for CpG group, n =5 for Dox-iRGD group, and n = 7 for the untreated group. Statistical analysis was performed using ANOVA with Fisher's LSD post hoc test. Source data are provided as a Source Data file.

a

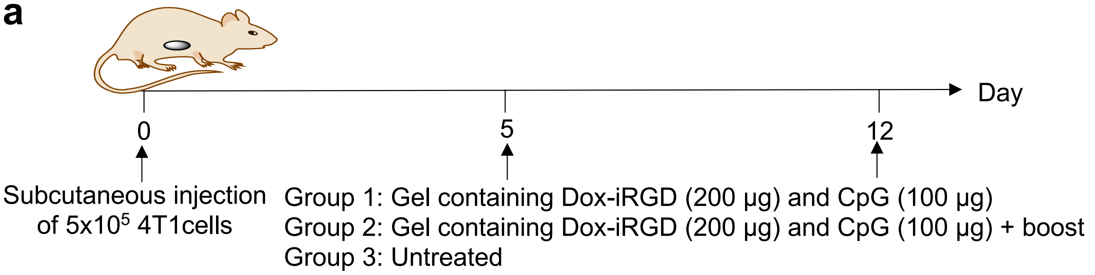

b

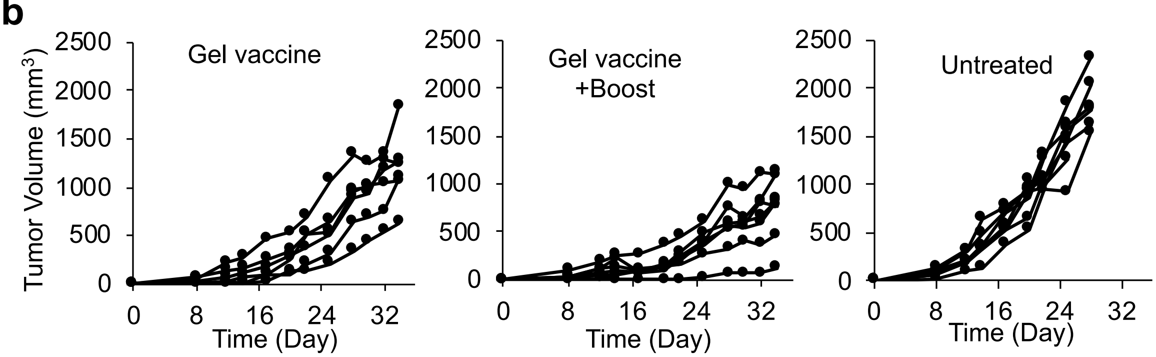

c

Group Description	Median Survival (day)	Survival Increase (day)	%Survival Increase
Gel	45.0	15.0	50.0
Gel + Boost	49.0	19.0	63.3
Untreated	30.0	--	--

d

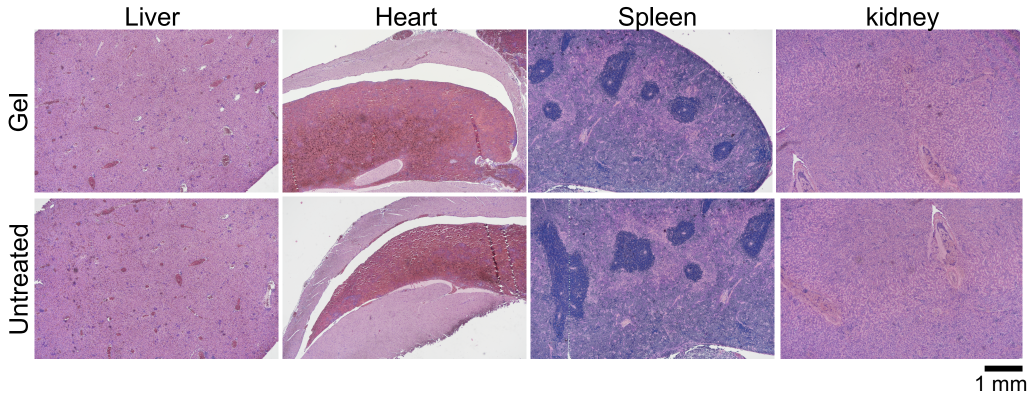

Supplementary Figure 10. **In situ gel vaccine containing GM-CSF, Dox-iRGD (200 µg) and CpG (100 µg) slows tumor growth and prolongs animal survival.** (a) Time frame of efficacy study. GM-CSF was incorporated in all gels. (b) Tumor growth curves for each animal of different groups. (c) Summary of median survival for each group and the increase of median survival in treatment groups in comparison to the untreated group. (d) Representative images of H&E stained liver, heart, spleen, and kidney tissues harvested from mice treated with the gel vaccine (upper row) and untreated mice (lower row). This experiment was repeated once.

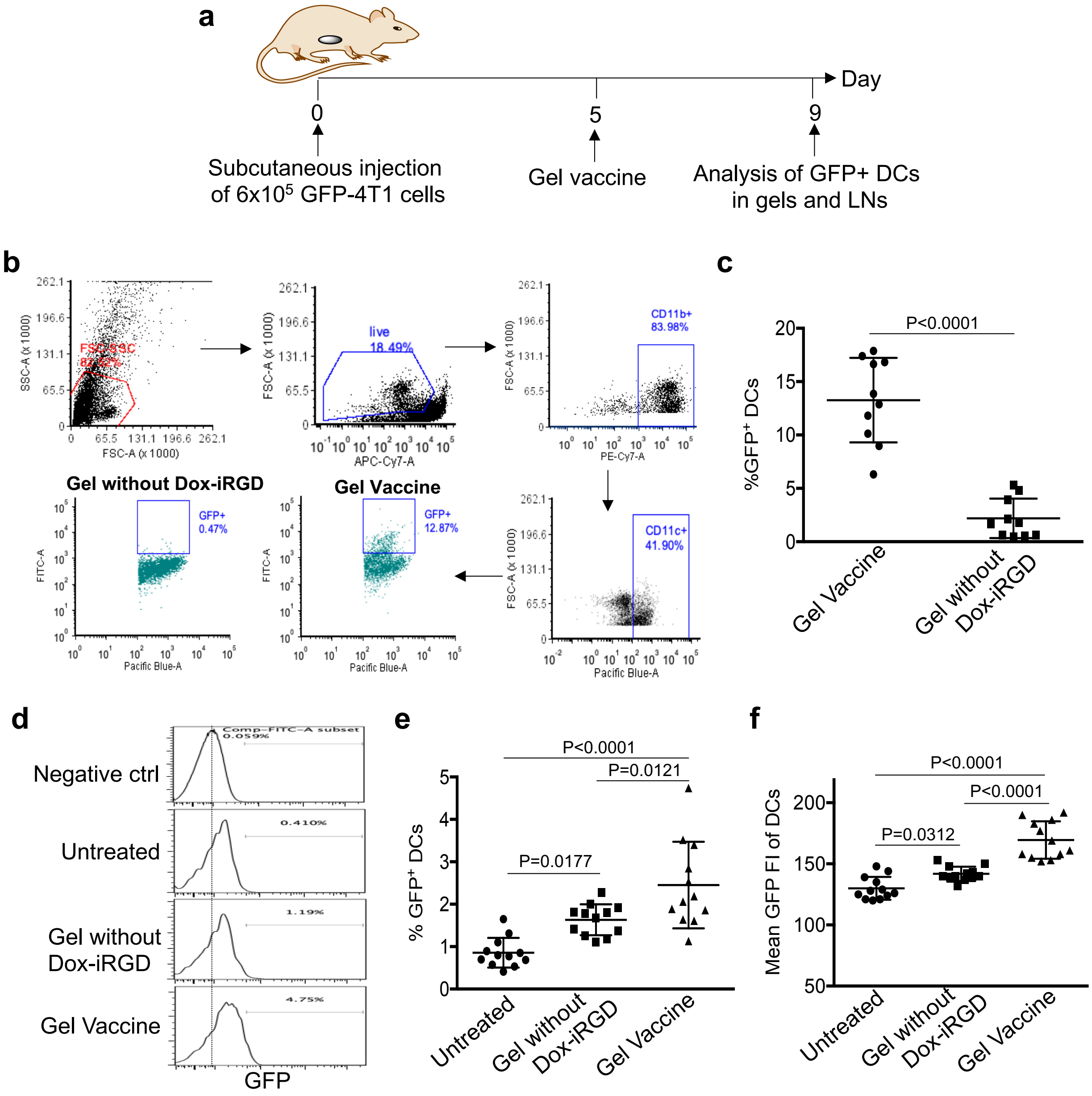

Supplementary Figure 11. Recruited DCs in the gel vaccines can sample tumor antigens. (a) Time frame of animal study. The full gel vaccine was loaded with GM-CSF, Dox-iRGD (200 µg), and CpG (100 µg). Gels loaded with GM-CSF and CpG were used as controls. (b) Representative flow cytometry profiles of cells isolated from the gel scaffolds. (c) Percentage of GFP^+ DCs ($CD11b^+CD11c^+$) in the gel vaccine or control gel without incorporation of Dox-iRGD. Data are presented as mean ± SD; n = 10 biologically independent animals per group. Statistical analysis was performed using a two-tailed t test. (d) Representative GFP histograms of cells isolated from tumor-draining lymph nodes (tdLNs). (e) Percentage of GFP^+ DCs in tdLNs. (f) Mean GFP fluorescence intensity of DCs in tdLNs. For e-f, data are presented as mean ± SD; n = 12 biologically independent animals per group. Statistical analysis was performed using ANOVA with Fisher's LSD post hoc test. Source data are provided as a Source Data file.

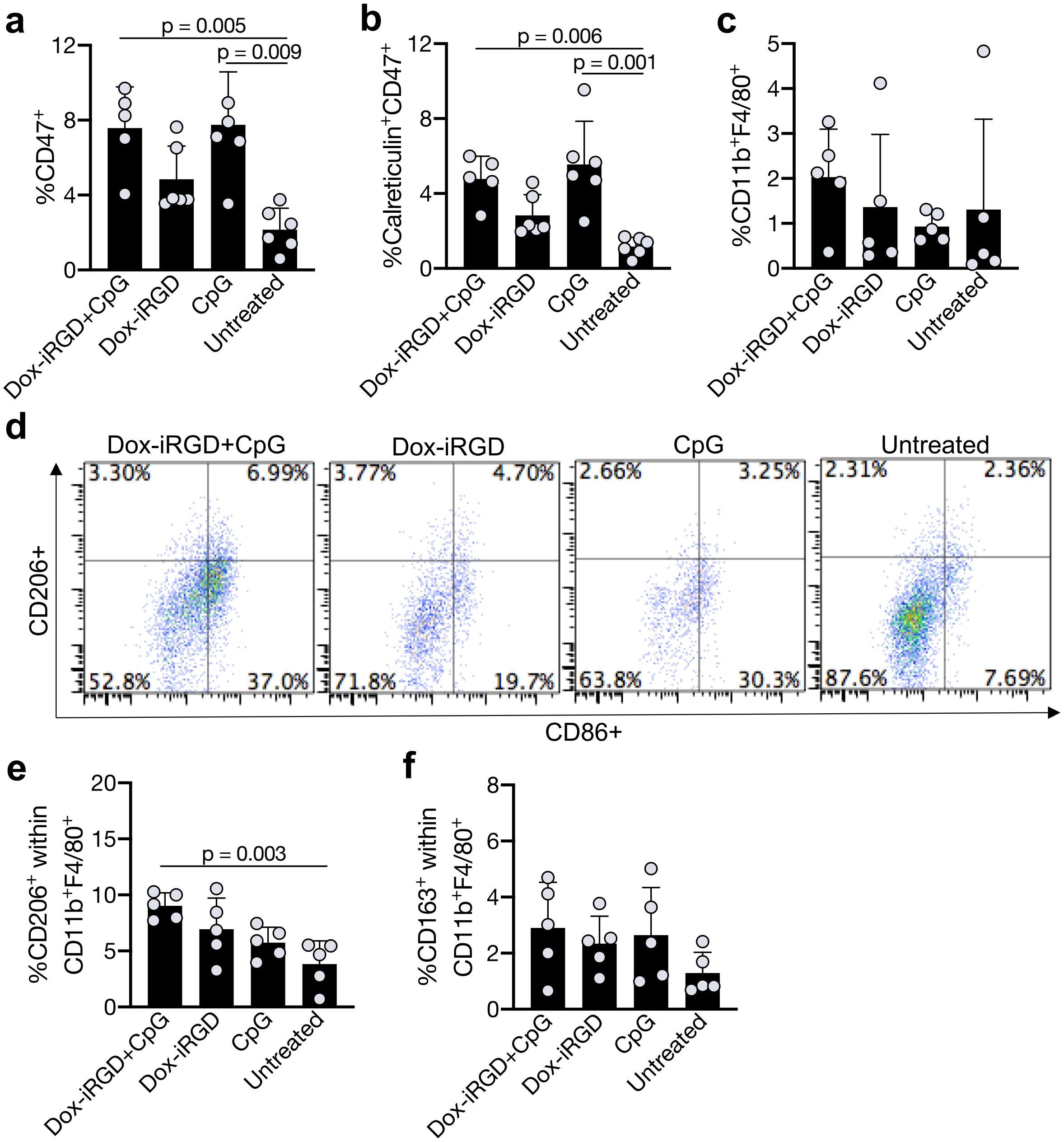

Supplementary Figure 12. **In situ gel vaccine induces immunogenic death of 4T1 cells and polarizes tumor-associated macrophages towards M1 phenotype.** Percentage of (a) $CD47^+$ and (b) $calreticulin^+CD47^+$ tumor cells at 11 days post injection of gels containing Dox-iRGD (200 μg) and CpG (100 μg) or Dox-iRGD alone or CpG alone. GM-CSF was incorporated in all gels. (c) Percentage of $CD11b^+F4/80^+$ macrophages in the tumor microenvironment. (d) Representative CD206 versus CD86 plots of tumor-associated macrophages in different groups. Percentage of (e) $CD206^+$ and (f) $CD163^+$ macrophages in tumors after different treatments. All numerical data are presented as mean ± SD; n = 6 biologically independent animals per group. For a-c, statistical analysis was performed using Kruskal-Wallis with Dunn's post hoc test. For e-f, statistical analysis was performed using ANOVA with Tukey's post hoc test. Source data are provided as a Source Data file.

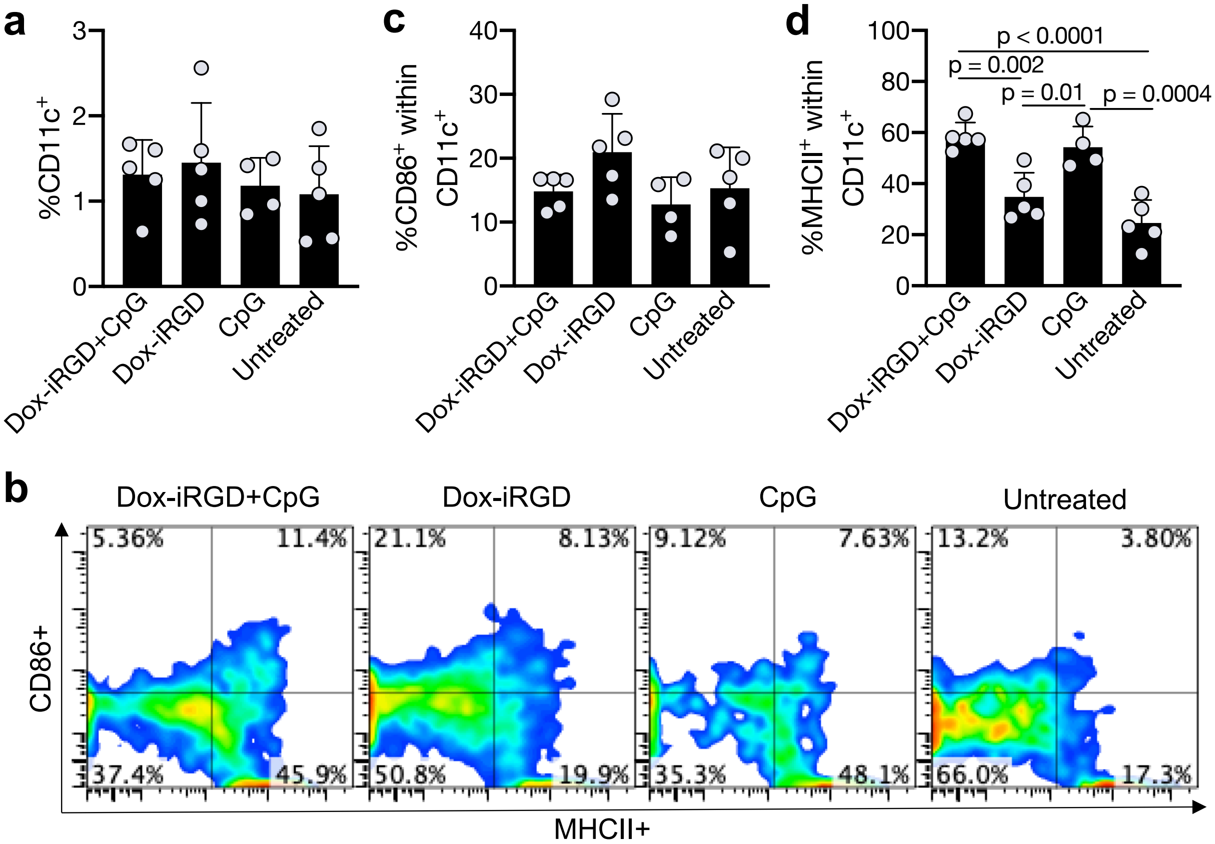

Supplementary Figure 13. In situ gel vaccine activates DCs in the tumor microenvironment. (a) Percentage of CD11c$^+$ DCs in tumors at 11 days post injection of gels containing Dox-iRGD (200 µg) and CpG (100 µg) or Dox-iRGD alone or CpG alone. GM-CSF was incorporated in all gels. (b) Representative CD86 versus MHC II plots of intratumoral DCs in different groups. Percentage of (c) CD86$^+$ DCs and (d) CD86$^+$MHCII$^+$ DCs in tumors after different treatments. For a and c-d, data are presented as mean ± SD; n = 6 biologically independent animals per group. Statistical analysis was performed using ANOVA with Tukey's post hoc test. Source data are provided as a Source Data file.

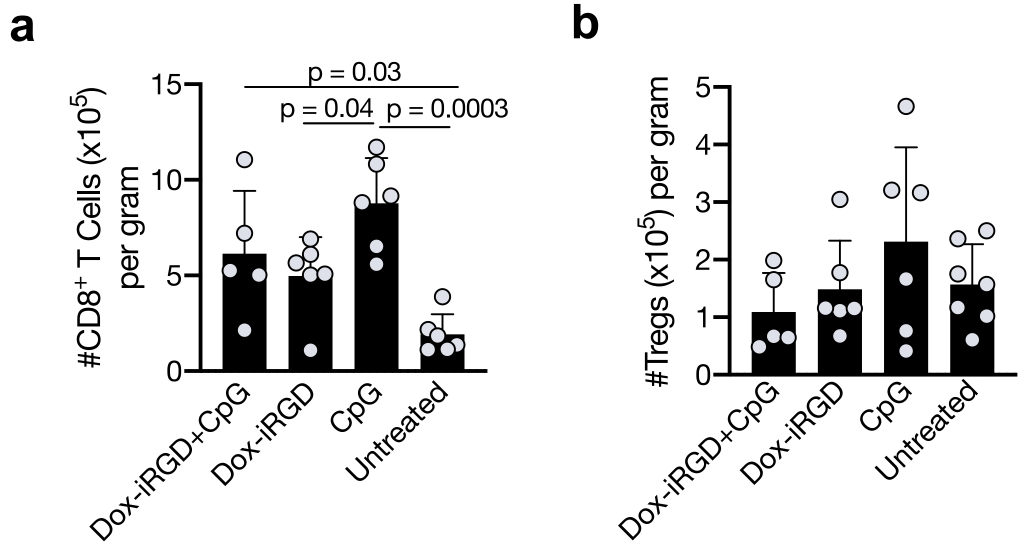

Supplementary Figure 14. **In situ gel vaccine increases tumor-infiltrating T cells**. Number of (a) tumor-infiltrating $CD8^+$ T cells and (b) $CD4^+FoxP3^+$ regulatory T cells at 11 days post injection of gels containing Dox-iRGD (200 µg) and CpG (100 µg) or Dox-iRGD alone or CpG alone. GM-CSF was incorporated in all gels. For a-b, data are presented as mean ± SD; n = 6 biologically independent animals per group. Statistical analysis was performed using ANOVA with Tukey's post hoc test. Source data are provided as a Source Data file.

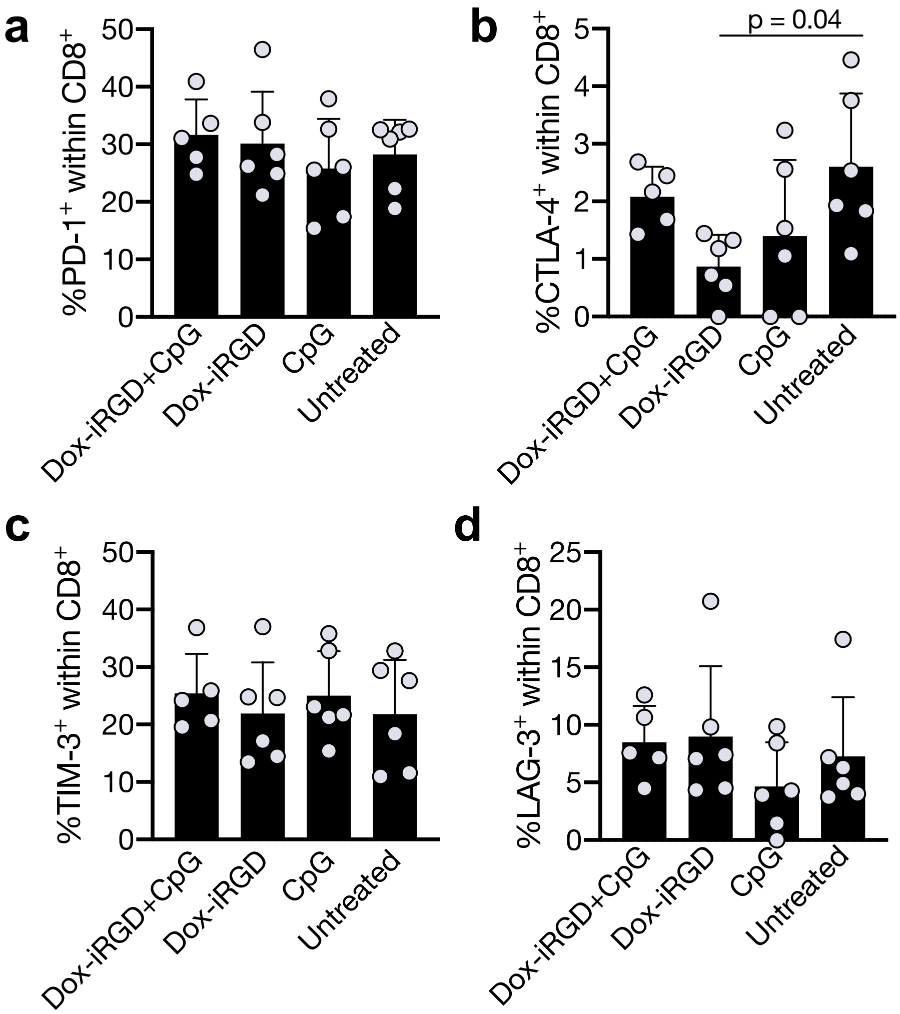

Supplementary Figure 15. **4T1 tumor-infiltrating CD8$^+$ T cells express exhaustion markers**. Percentage of (a) PD-1$^+$, (b) CTLA-4$^+$, (c) TIM3$^+$, and (d) LAG3$^+$ cells among intratumoral CD8$^+$ T cells at 11 days post injection of gels containing Dox-iRGD (200 µg) and CpG (100 µg) or Dox-iRGD alone or CpG alone. GM-CSF was incorporated in all gels. For a-d, data are presented as mean ± SD; n = 6 biologically independent animals per group. For b, statistical analysis was performed using ANOVA with Tukey's post hoc test. Source data are provided as a Source Data file.

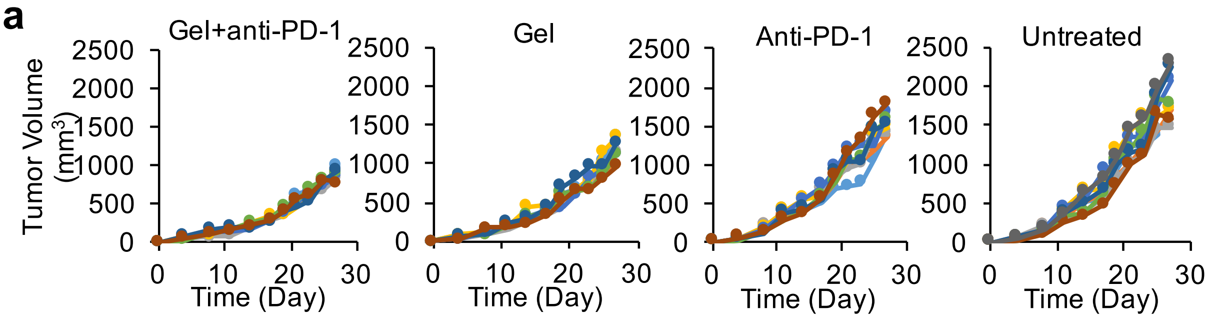

b

Group Description	Median Survival (day)	Survival Increase (day)	%Survival Increase
Gel+anti-PD-1	40	13	48.1
Gel	37.5	10.5	38.9
Anti-PD-1	28	1	3.7
Untreated	27	--	--

Supplementary Figure 16. **In situ gel vaccine synergizes with anti-PD-1 therapy for tumor control**. (a) Tumor growth curves for each animal of different groups. (b) Summary of median survival for each group and the increase of median survival in treatment groups in comparison to the untreated group.

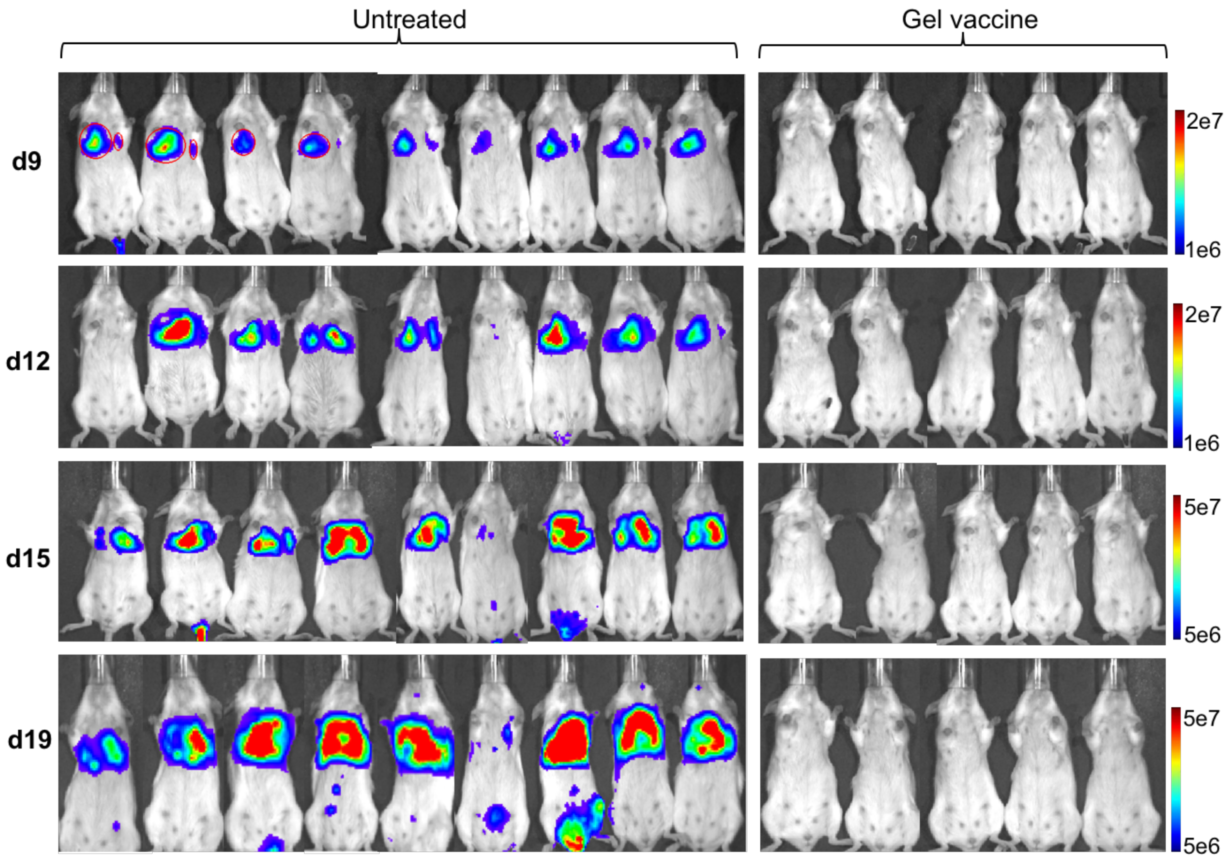

Supplementary Figure 17. In situ gel vaccines injected at the tumor resection site prevent the formation of 4T1 metastatic cancers. Following surgical resection of 4T1 tumors, gels containing GM-CSF, Dox-iRGD (200 μg) and CpG (100 μg) were injected at the surgical site. Mice were re-challenged with i.v. injected luciferase-expressing 4T1 (luc-4T1) cells at ~80 days post gel injection. Shown are bioluminescence images of mice at different times post i.v. injection of luc-4T1 cells.

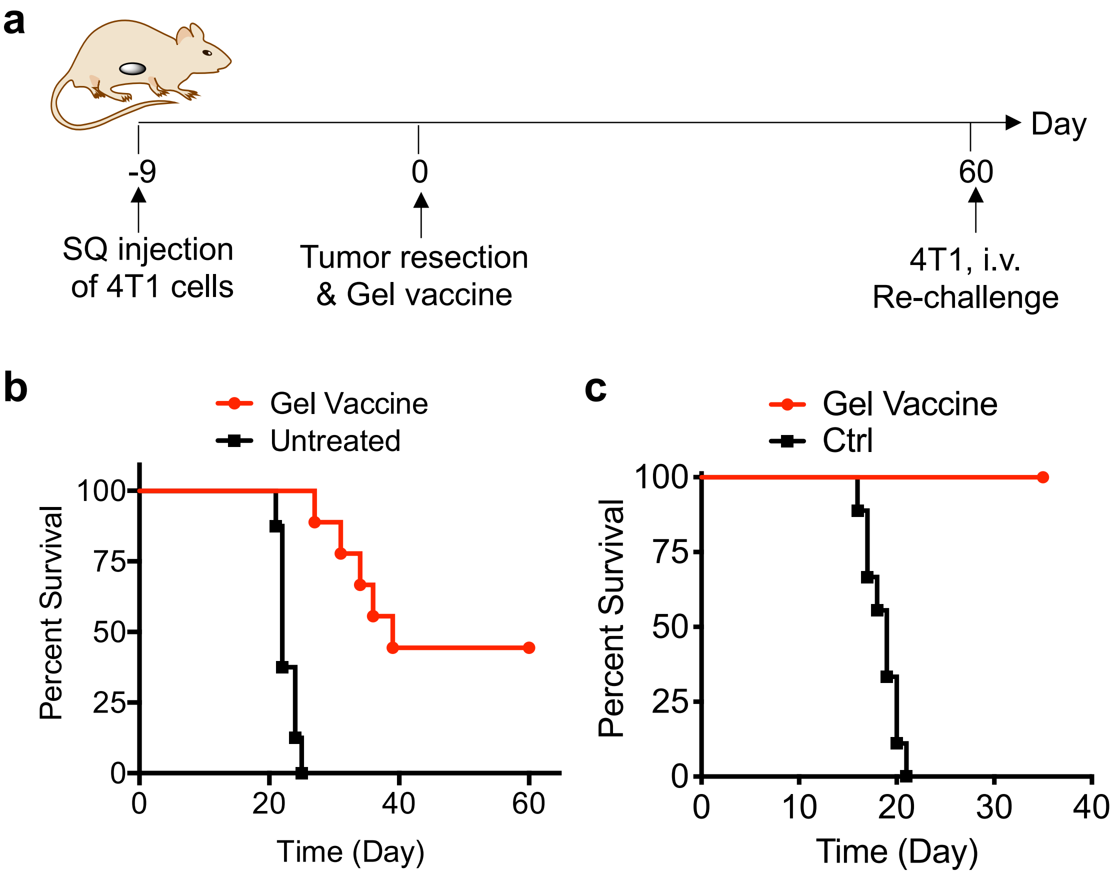

Supplementary Figure 18. **In situ gel vaccines injected at the tumor resection site prevent the formation of 4T1 metastatic cancers**. (a) Time frame of the animal study. Following surgical resection of 4T1 tumors, gels containing GM-CSF, Dox-iRGD (200 μg) and CpG (100 μg) were injected at tumor base. Mice were re-challenged with i.v. injected 4T1 cells at 60 days post gel injection. (b) Kaplan-Meier plots for overall survival of untreated mice or mice treated with the gel vaccine at tumor resection site. (c) Kaplan-Meier plots for overall survival of control mice (no previous treatment nor tumor inoculation) and mice that survived from (b), after 4T1 tumor challenge. For b-c, n = 9 biologically independent animals per group. Source data are provided as a Source Data file.

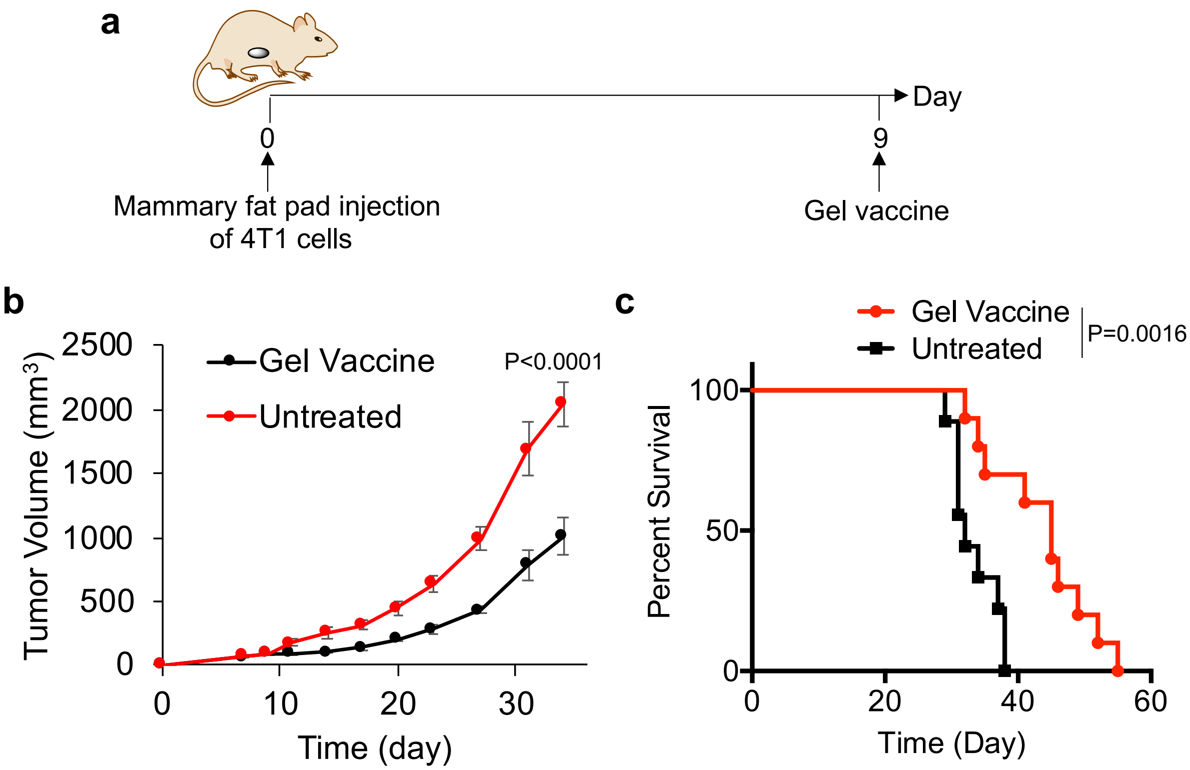

Supplementary Figure 19. **In situ gel vaccine slows tumor growth and prolongs animal survival in an orthotopic 4T1 tumor model**. (a) Time frame of the animal study. Gels were loaded with GM-CSF, Dox-iRGD (200 µg) and CpG (100 µg). (b) Tumor growth profiles for each group. Data are presented as mean ± SEM; statistical analysis was performed using two-tailed t tests. (c) Kaplan-Meier plots for overall survival of all groups. Statistical analysis was performed using the log-rank (Mantel-Cox) test, For b-c, n = 9 biologically-independent animals for untreated group and n=10 for gel vaccine group. Source data are provided as a Source Data file.

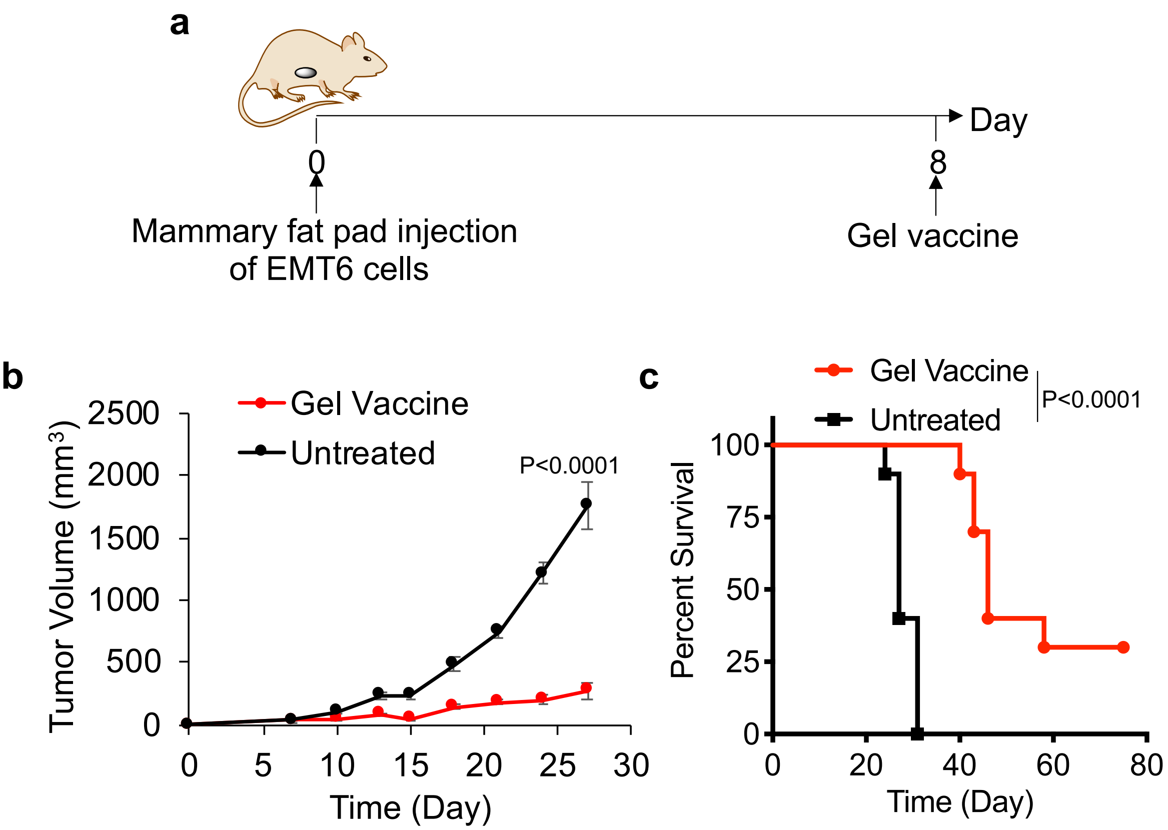

Supplementary Figure 20. **In situ gel vaccine slows tumor growth and prolongs animal survival in an orthotopic EMT6 tumor model**. (a) Time frame of the animal study. Gels were loaded with GM-CSF, Dox-iRGD (200 µg) and CpG (100 µg). (b) Tumor growth profiles for each group. Data are presented as mean ± SEM; statistical analysis was performed using two-tailed t tests. (c) Kaplan-Meier plots for overall survival of all groups. Statistical analysis was performed using the log-rank (Mantel-Cox) test, For b-c, n = 10 biologically-independent animals per group. Source data are provided as a Source Data file.

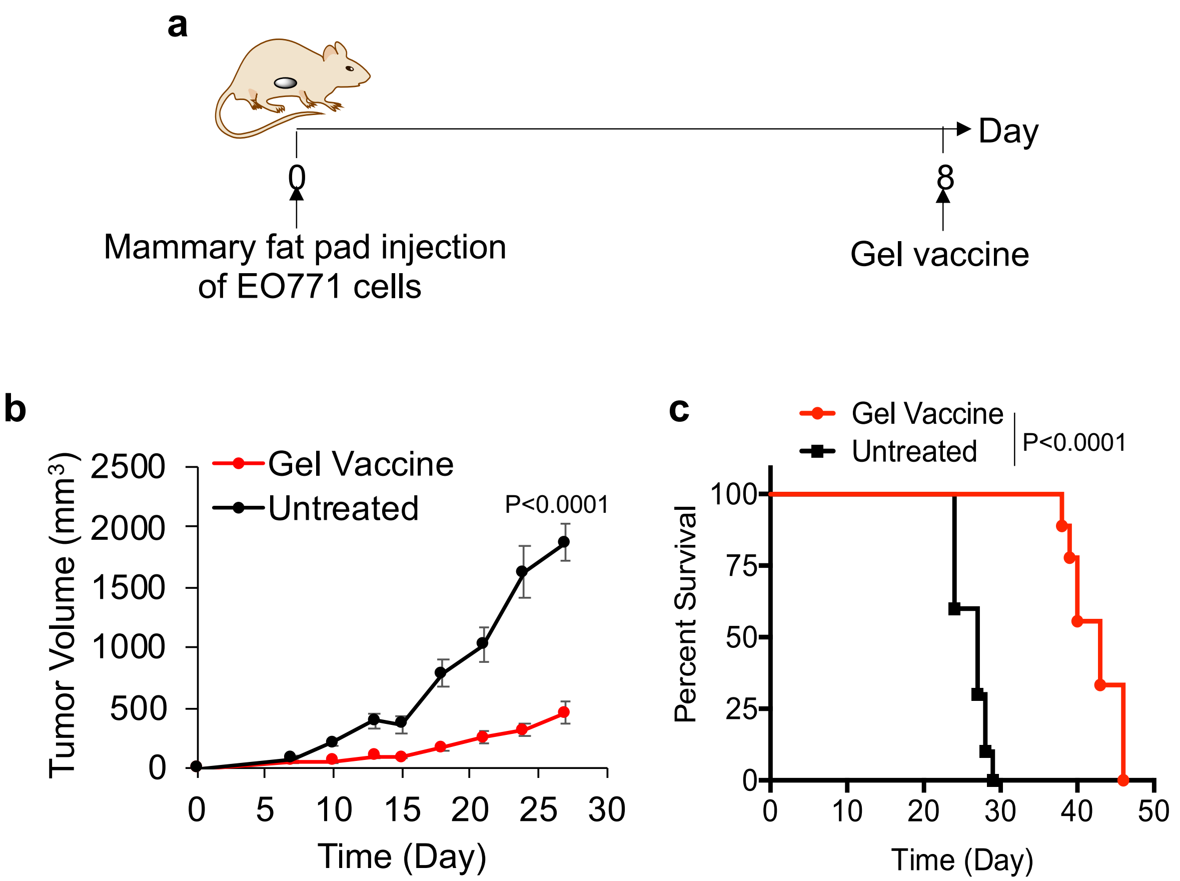

Supplementary Figure 21. In situ gel vaccine slows tumor growth and prolongs animal survival in an orthotopic EO771 tumor model. (a) Time frame of the animal study. Gels were loaded with GM-CSF, Dox-iRGD (200 µg) and CpG (100 µg). (b) Tumor growth profiles for each group. Data are presented as mean ± SEM; statistical analysis was performed using two-tailed t tests. (c) Kaplan-Meier plots for overall survival of all groups. Statistical analysis was performed using the log-rank (Mantel-Cox) test, For b-c, n = 9 biologically-independent animals for gel vaccine group and n=10 for untreated group. Source data are provided as a Source Data file.

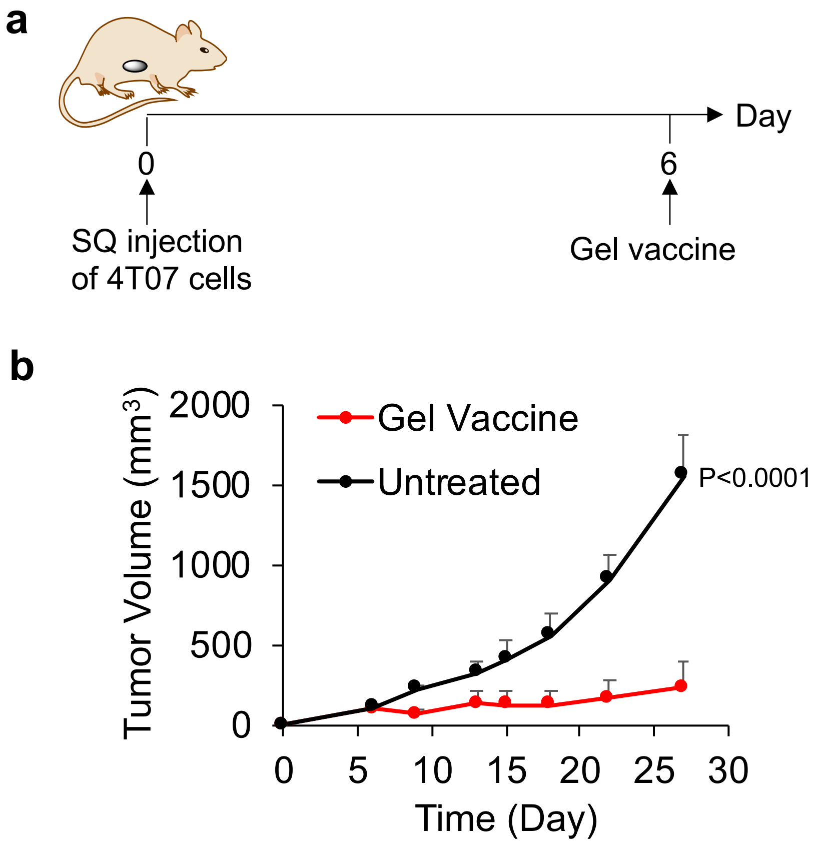

Supplementary Figure 22. **In situ gel vaccine slows potent efficacy against 4T07 tumors**. (a) Time frame of the animal study. Gels were loaded with GM-CSF, Dox-iRGD (200 μg) and CpG (100 μg). (b) Tumor growth profiles for each group. Data are presented as mean ± SEM; n = 10 biologically-independent animals per group. Statistical analysis was performed using two-tailed t tests. Source data are provided as a Source Data file.

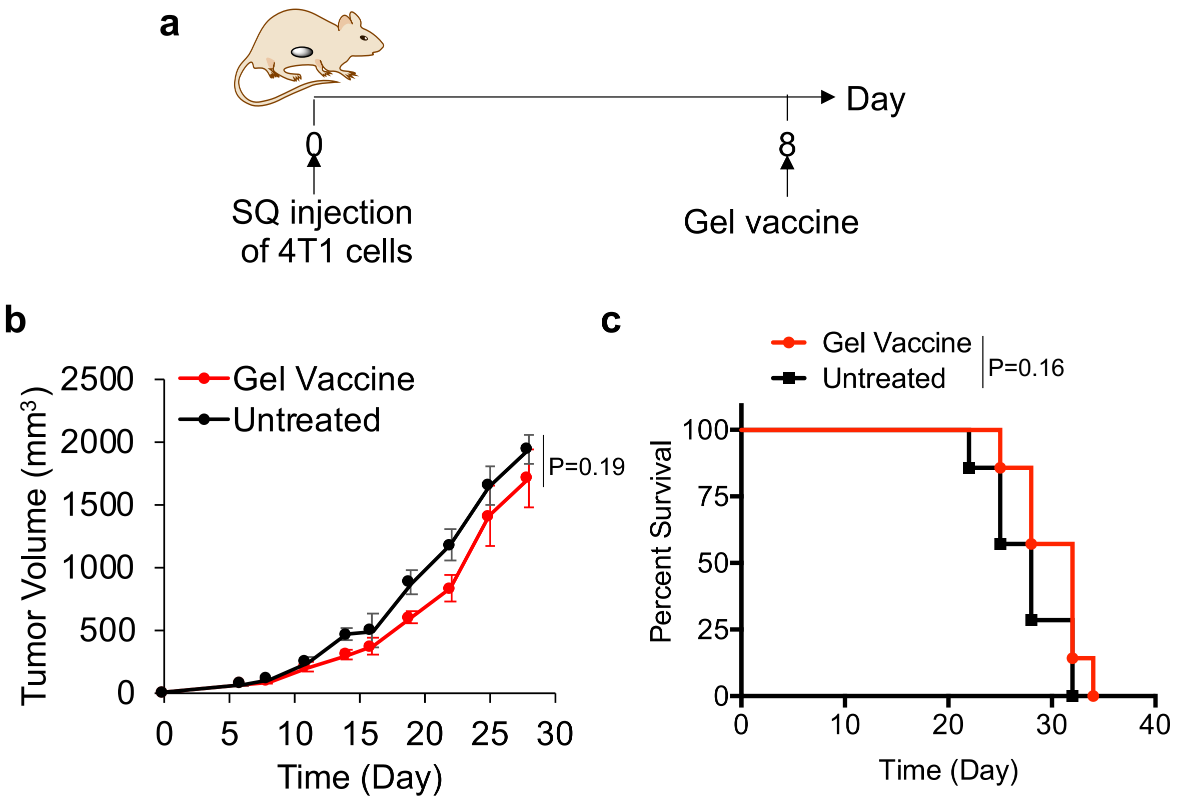

Supplementary Figure 23. **In situ gel vaccine does not show therapeutic benefit against 4T1 tumors in athymic nude mice**. (a) Time frame of the animal study. Gels were loaded with GM-CSF, Dox-iRGD (200 µg) and CpG (100 µg). Athymic NU/J mice were used. (b) Tumor growth profiles for each group. Data are presented as mean ± SD; statistical analysis was performed using two-tailed t tests. (c) Kaplan-Meier plots for overall survival of all groups. Statistical analysis was performed using the log-rank (Mantel-Cox) test, For b-c, n = 7 biologically-independent animals per group. Source data are provided as a Source Data file.

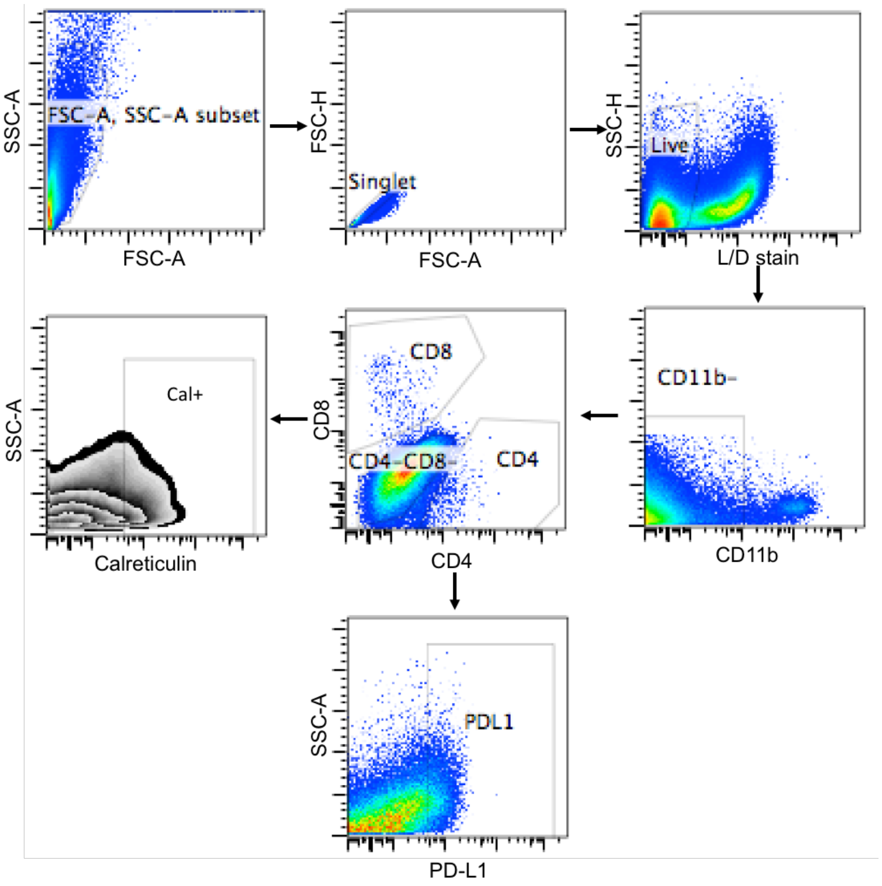

Supplementary Figure 24. **Representative gating strategy for calreticulin- or PD-L1-positive tumor cells.** Singlet cells were selected from the cell population and dead cells were excluded. $CD11b^{+}$, $CD4^{+}$, and $CD8^{+}$ cells were excluded prior to analyses of tumoral calreticulin and PD-L1 levels.

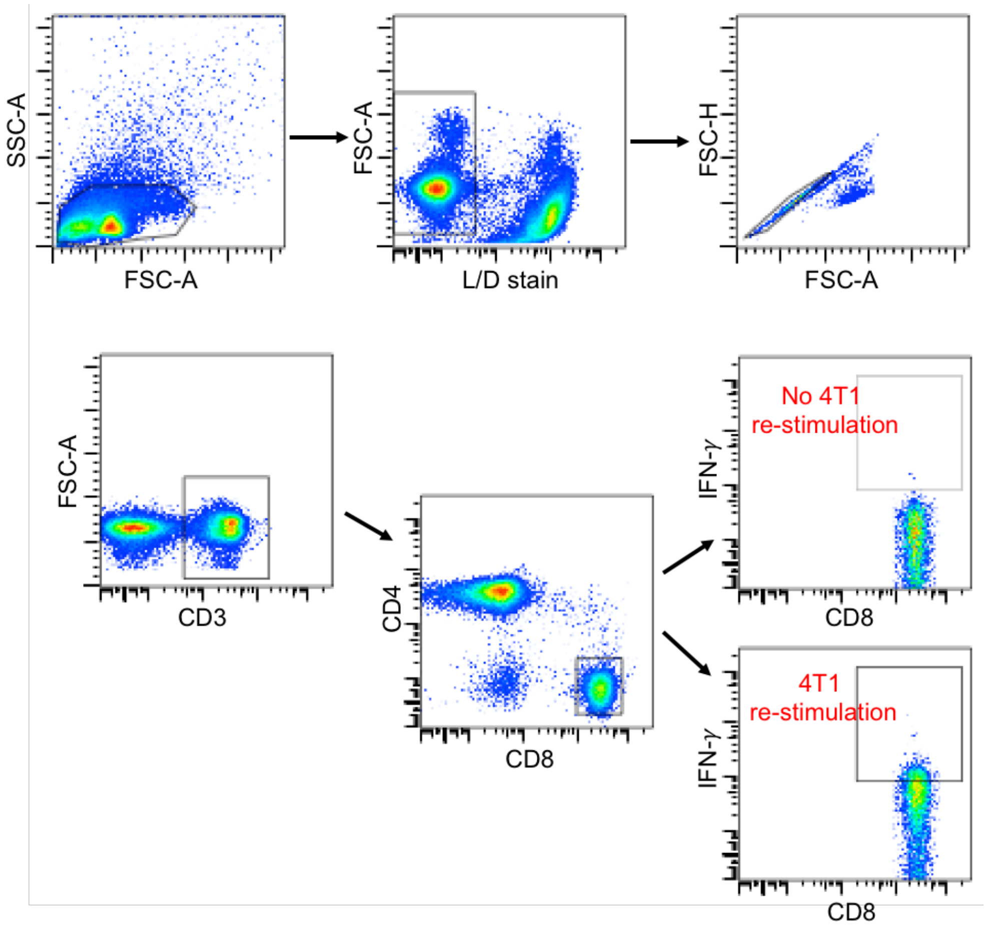

Supplementary Figure 25. Representative gating strategy for IFN-γ^+ CD8$^+$ T cells in lymph nodes or spleens. Dead cells were excluded and singlet cells were selected from the cell population. CD3$^+$CD8$^+$ T cells were then selected for analyses of IFN-γ expression. Representative plots of cell suspensions with or without in vitro re-stimulation with 4T1 cells are shown.
