# Supplementary material for: Biomaterial-based scaffold for in situ chemo-immunotherapy to treat poorly immunogenic tumors
Source: Nat Commun. 2020 Nov 10;11:5696. doi: 10.1038/s41467-020-19540-z (PMC7655953; doi:10.1038/s41467-020-19540-z)
Supplement: Supplementary file 3 — Reporting Summary [file 41467_2020_19540_MOESM3_ESM.pdf]

nature research

Corresponding author(s): David J. Mooney

Last updated by author(s): 10/1/2020

Reporting Summary

Nature Research wishes to improve the reproducibility of the work that we publish. This form provides structure for consistency and transparency in reporting. For further information on Nature Research policies, see our Editorial Policies and the Editorial Policy Checklist.

Statistics

For all statistical analyses, confirm that the following items are present in the figure legend, table legend, main text, or Methods section.

n/a	Confirmed	
[ ]	[x]	The exact sample size (*n*) for each experimental group/condition, given as a discrete number and unit of measurement
[ ]	[x]	A statement on whether measurements were taken from distinct samples or whether the same sample was measured repeatedly
[ ]	[x]	The statistical test(s) used AND whether they are one- or two-sided<br>*Only common tests should be described solely by name; describe more complex techniques in the Methods section.*
[x]	[ ]	A description of all covariates tested
[ ]	[x]	A description of any assumptions or corrections, such as tests of normality and adjustment for multiple comparisons
[ ]	[x]	A full description of the statistical parameters including central tendency (e.g. means) or other basic estimates (e.g. regression coefficient) AND variation (e.g. standard deviation) or associated estimates of uncertainty (e.g. confidence intervals)
[ ]	[x]	For null hypothesis testing, the test statistic (e.g. *F*, *t*, *r*) with confidence intervals, effect sizes, degrees of freedom and *P* value noted<br>*Give P values as exact values whenever suitable.*
[x]	[ ]	For Bayesian analysis, information on the choice of priors and Markov chain Monte Carlo settings
[x]	[ ]	For hierarchical and complex designs, identification of the appropriate level for tests and full reporting of outcomes
[x]	[ ]	Estimates of effect sizes (e.g. Cohen's *d*, Pearson's *r*), indicating how they were calculated

Our web collection on statistics for biologists contains articles on many of the points above.

Software and code

Policy information about availability of computer code

Data collection	Flow cytometry data were collected via BD FACS Diva on either BD LSRII or BD LSR Fortessa flow cytometers
Data analysis	Flow cytometry data were analyzed on FlowJo v7.6 and FCSExpress v6 and v7. Statistical testing was performed using GraphPad Prism v6 and v8 and Microsoft Excel (v16.25).

For manuscripts utilizing custom algorithms or software that are central to the research but not yet described in published literature, software must be made available to editors and reviewers. We strongly encourage code deposition in a community repository (e.g. GitHub). See the Nature Research guidelines for submitting code & software for further information.

Data

Policy information about availability of data

All manuscripts must include a data availability statement. This statement should provide the following information, where applicable:

- Accession codes, unique identifiers, or web links for publicly available datasets
- A list of figures that have associated raw data
- A description of any restrictions on data availability

All data supporting the results are provided with the manuscript. Raw data are publicly available (https://doi.org/10.7910/DVN/N3I4BQ) and available from the corresponding author (mooneyd@seas.harvard.edu) upon reasonable request, as stated in the data availability statement within the manuscript. The public repository will be made accessible the day of publication.

Field-specific reporting

Please select the one below that is the best fit for your research. If you are not sure, read the appropriate sections before making your selection.

- [x] Life sciences
- [ ] Behavioural & social sciences
- [ ] Ecological, evolutionary & environmental sciences

For a reference copy of the document with all sections, see nature.com/documents/nr-reporting-summary-flat.pdf

Life sciences study design

All studies must disclose on these points even when the disclosure is negative.

Sample size	Sample sizes of 3-8 biologically independent samples per group and 4-10 biologically independent animals per group were used for in vitro and animal studies, respectively, as indicated for specific experiments in Figure captions. Sample sizes for in vivo studies were determined empirically based on results from prior publications to achieve statistical power balanced with input and approval from Harvard University's Institutional Animal Care and Use Committee.
Data exclusions	No data were excluded.
Replication	All key studies were successfully replicated at least once. Supplemental experiments added in response to reviewer suggestions were performed once.
Randomization	Animals were randomly allocated to different groups before treatment. For in vitro experiments, randomization was not necessary as samples were tested in well-controlled conditions.
Blinding	In vitro experiments were unblinded as all samples were processed identically in a well-controlled setting. The initial in vivo monitoring and survival experiments were conducted in an unblinded manner, to first establish a proof-of-principle of whether there was a therapy-mediated enhancement in survival that could be further explored in a blinded setting. Subsequent repeat survival experiments were conducted by researchers who were blinded to the treatment.

Reporting for specific materials, systems and methods

We require information from authors about some types of materials, experimental systems and methods used in many studies. Here, indicate whether each material, system or method listed is relevant to your study. If you are not sure if a list item applies to your research, read the appropriate section before selecting a response.

Materials & experimental systems

n/a	Involved in the study	
[ ]	[x]	Antibodies
[ ]	[x]	Eukaryotic cell lines
[x]	[ ]	Palaeontology and archaeology
[ ]	[x]	Animals and other organisms
[x]	[ ]	Human research participants
[x]	[ ]	Clinical data
[x]	[ ]	Dual use research of concern

Methods

n/a	Involved in the study	
[x]	[ ]	ChIP-seq
[ ]	[x]	Flow cytometry
[x]	[ ]	MRI-based neuroimaging

Antibodies

Antibodies used	Primary antibodies used in this study include anti-PD-1 (RMP1-14, InVivoMAb anti-mouse PD-1, BioXCell), brilliant violet 421-conjugated anti-CD11b (M1/70, Biolegend #101235), FITC-conjugated anti-CD11c (N418, Biolegend #117306), PE/Cy7-conjugated anti-CD3 (145-2C11, Biolegend #100320), PE-conjugated anti-F4/80 (BM8.1, Tonbo biosciences #50-4801), APC-conjugated anti-Gr1 (RB6-8C5, Biolegend #108411), PE/Cy7-conjugated anti-MHCII (M5/114.15.2, ebioscience #25-5321-82), and PE-conjugated anti-CD86 (GL1, ebioscience #12-0862-81), efluor450-conjugated anti-CD4 (RM4-5, ebioscience #48-0042-82), and FITC-conjugated anti-CD8 (53-6.7, Biolegend #100706), APC-conjugated IFN- (XMG1.2, ebioscience #17-7311-82) and PE-conjugated anti-TNF- (MP6-XT22 , ebioscience # 12-7321-41), pacific blue-conjugated anti-CD103 (2E7, Biolegend #121418), PerCP/Cy5.5-conjugated anti-PD-L1 (10F.9G2, Biolegend #124334), APC-conjugated anti-CD8 (53-6.7, Biolegend #100712), PE/Cy7-conjugated anti-CD11b (M1/70, ebioscience #25-0112-81), efluor450-conjugated anti-CD163 (TNKUPJ, Biolegend # 48-1631-82), APC-conjugated anti-CD206 (C068C2, Biolegend #141707), FITC-conjugated anti-CD86 (GL-1, Biolegend #105006), efluor450-conjugated anti-CD8 (53-6.7, ebioscience #48-0081-82), FITC-conjugated anti-PD-1 (29F.1A12, Biolegend #135214), PE-conjugated anti-LAG3 (C9B7W, Biolegend #125207), APC-conjugated anti-CTLA-4 (L3D10, Biolegend #349907), PE/Cy7-conjugated anti-TIM-3 (RMT3-23, Biolegend #119715), FITC-conjugated anti-CD25 (PC61, Biolegend #100206), PE-conjugated anti-Foxp3 (MF-14, Biolegend #126404), Alexa fluor 647-conjugated anti-calreticulin (EPR3924, Abcam #ab196159), and FITC-conjugated anti-CD47 (miap301, Biolegend #127504). Fixable viability dye efluor780 was obtained from Thermo Fisher Scientific (#65-0865-14).. All antibodies were diluted according to manufacturer recommendations.

Validation	All antibodies were purchased from the supplier as noted above and validated by the suppliers. No further validation was performed. Supplier validation included quality control testing by immunofluorescent staining with flow cytometric analysis on sample cells. For examples, see: https://www.biolegend.com/en-us/products/fitc-anti-mouse-cd11c-antibody-1815, https://www.thermofisher.com/antibody/product/CD8a-Antibody-clone-53-6-7-Monoclonal/48-0081-82.

Eukaryotic cell lines

Policy information about cell lines

Cell line source(s)	The following cell lines were used: 4T1 cell line (ATCC), 4T07 cell line (Karmanos Cancer Institute), 4T1-eGFP-Puro cell line (Imanis Life Sciences), EMT-6 cell line (ATCC), EO771 cell line (gift from Dana Farber Cancer Institute), as listed in the methods section of the manuscript.
Authentication	Cell line authentication was conducted following ATCC recommendations by checking consistency of cell morphology by bright-field microscopy and growth curve analysis (http://www.atcc.org/Services/Testing Services/Cell Authentication Testing Service/Cell Line Authentication Test Recommendations.aspx)
Mycoplasma contamination	Cells were not tested for mycoplasma contamination.
Commonly misidentified lines (See ICLAC register)	No commonly misidentified cell lines were used in this study.

Animals and other organisms

Policy information about studies involving animals; ARRIVE guidelines recommended for reporting animal research

Laboratory animals	Female BALB/c mice were purchased from the Jackson Laboratory (Bar Harbor, ME, USA) and were 6-8 weeks old at the beginning of each experiment. Feed and water were available ad libitum. Artificial light was provided in a 12 h/12 h cycle.
Wild animals	The study did not involve wild animals.
Field-collected samples	The study did not involve field-collected samples.
Ethics oversight	Harvard University Institutional Animal Care and Use Committee

Note that full information on the approval of the study protocol must also be provided in the manuscript.

Flow Cytometry

Plots

Confirm that:

- [x] The axis labels state the marker and fluorochrome used (e.g. CD4-FITC).
- [x] The axis scales are clearly visible. Include numbers along axes only for bottom left plot of group (a 'group' is an analysis of identical markers).
- [x] All plots are contour plots with outliers or pseudocolor plots.
- [x] A numerical value for number of cells or percentage (with statistics) is provided.

Methodology

Sample preparation	For flow cytometry analysis of cells, blood samples were red-blood-cell-lysed and stained with antibodies. Tissue (bone marrow, spleen) was harvested, homogenized, and a single cell suspension was prepared. Antibodies were diluted according to the manufacturer's suggestions.
Instrument	BD LSR Fortessa or BD LSRII
Software	Collect: BD FACSDiva; analysis: FlowJo v7.6 and FCSExpress v6 and v7.
Cell population abundance	At least 20,000 total events were acquired for all FACS analyses.
Gating strategy	All cells were gated based on forward-scatter and side-scatter characteristics to limit debris including dead cells. Cells were gated based on positive and fluorescence minus one controls, and the frequencies of cells staining positive for each marker was recorded.

- [x] Tick this box to confirm that a figure exemplifying the gating strategy is provided in the Supplementary Information.
